# The Use of Chinese Yang/Qi-Invigorating Tonic Botanical Drugs/Herbal Formulations in Ameliorating Chronic Kidney Disease by Enhancing Mitochondrial Function

**DOI:** 10.3389/fphar.2021.622498

**Published:** 2021-06-24

**Authors:** Jiayi Tian, Yuqi Huang, Tong Wu, Hsien-Da Huang, Kam Ming Ko, Bao Ting Zhu, Jihang Chen

**Affiliations:** ^1^ Shenzhen Key Laboratory of Steroid Drug Discovery and Development, School of Life and Health Sciences, The Chinese University of Hong Kong, Shenzhen, China; ^2^ School of Life and Health Sciences, The Chinese University of Hong Kong, Shenzhen, China; ^3^ Warshel Institute for Computational Biology, The Chinese University of Hong Kong, Shenzhen, China; ^4^ Division of Life Science, The Hong Kong University of Science & Technology, Hong Kong, China

**Keywords:** Yang/Qi invigoration, Chinese medicine, mitochondrial function, mitochondrial antioxidant capacity, chronic kidney disease

## Abstract

**Background:** Chronic kidney disease (CKD) is a leading cause of morbidity and mortality. Mitochondrial dysfunction has been implicated as a key factor in the development of CKD. According to traditional Chinese medicine (TCM) theory, many Chinese Yang/Qi-invigorating botanical drugs/herbal formulations have been shown to produce promising outcomes in the clinical management of CKD. Experimental studies have indicated that the health-promoting action of Yang/Qi invigoration in TCM is related to the up-regulation of mitochondrial energy generation and antioxidant status.

**Objective:** In this review, we aim to test whether Chinese Yang/Qi-invigorating tonic botanical drugs/herbal formulations can provide medical benefits in CKD and its complications. And we also explore the possible involvement of mitochondrial-associated signaling pathway underlying the beneficial effects of Yang/Qi invigoration in TCM.

**Methods:** A systematic search of “PubMed”, “China National Knowledge Infrastructure (CNKI)” and “Google Scholar” was carried out to collect all the available articles in English or Chinese related to Chinese Yang/Qi-invigorating tonic botanical drugs/herbal formulations and their effects on mitochondrial function and chronic kidney disease.

**Result and Discussion:** The relationship between the progression of CKD and mitochondrial function is discussed. The effects of Chinese Yang/Qi-invigorating tonic botanical drugs/herbal formulations and their active ingredients, including phytosterols/triterpenes, flavonoids, and dibenzocyclooctadiene lignans, on CKD and related alterations in mitochondrial signaling pathways are also presented in this review. In the future, exploration of the possible beneficial effects and clinical studies of more Yang- and Qi-invigorating botanical drugs/herbal formulations in the prevention and/or/treatment of CKD and the molecular mechanisms relating to the enhancement of mitochondrial functions warrants further investigation.

**Conclusion:** Given the critical role of mitochondrial function in safeguarding renal functional integrity, the enhancement of mitochondrial energy metabolism and antioxidant status in kidney tissue is likely involved in renal protection. Future studies on the biochemical and chemical basis underlying the effects of Chinese Yang/Qi-invigorating tonic botanical drugs/herbal formulations from a mitochondrial perspective will hopefully provide novel insights into the rational development of new drugs for the prevention and/or treatment of CKD.

## Introduction

Chronic kidney disease (CKD) is a global health problem, which poses a great threat to a growing number of people worldwide. A systematic analysis of CKD showed that its global prevalence is 9.1%, with 1.2 million deaths having resulted from CKD and its complications in 2017. (Theo and Boris, 2017). CKD is a long-term clinical condition characterized by impaired renal structure and function, as well as a reduction in glomerular filtration rate. Although the etiology of CKD is complex, mitochondrial dysfunction has been widely accepted as a major contributing factor in its pathogenesis (Takemura et al., 2020). Mitochondrial dysfunction results in defective mitochondrial oxidative phosphorylation, a reduction in ATP production, and an increase in mitochondrial DNA damage, all of which contribute to the progression of the disease. A growing body of experimental and clinical evidence has demonstrated that mitochondria-targeted therapeutic agents may offer a promising approach to the alleviation of CKD-related disease (Galvan et al., 2017).

Traditional Chinese medicine (TCM) has long been used as an integral part of mainstream medicine for the treatment of CKD in China. Based on TCM theory, the pathogenesis of CKD is complex, in that CKD is considered to be the consequence of an assault from both internal and external “evils” (i.e., pathogenic factors), which further leads to the imbalance of Yin and Yang function in the body (Gobe and Shen, 2015). According to clinical observations in patients suffering from CKD, the most common syndrome or disease pattern in TCM is “Kidney-Yang” deficiency (Wang et al., 2016). In this regard, in the practice of TCM, many Chinese Yang/Qi-invigorating tonic botanical drugs/herbal formulations have been shown to ameliorate the symptoms of CKD. The exploration of these Yang/Qi tonic botanical drugs as the source of new agents for the treatment of CKD has recently attracted the increasing attention of many researchers (Zhong et al., 2013). For instance, You-Gui Pill, a typical Yang/Qi-tonic formulation, possesses the kidney yang-tonifying effects according to the TCM theory. It has been clinically used as the basic formula for the treatment of patients with Kidney-Yang Deficiency syndrome for hundreds of years in China. A clinical study in China showed that treatment of You-Gui Pill can significantly lower serum creatinine levels, reduce proteinuria and improve glomerular filtration in CKD patients with yang-deficiency syndrome (Liu et al., 2016). However, the biochemical and molecular mechanisms underlying the possible therapeutic effects of Yang/Qi-invigorating tonic botanical drugs on CKD remain largely unknown. Results obtained from experimental studies indicate that the health-promoting effect of Yang/Qi-invigoration in TCM is closely related to the up-regulation of mitochondrial function and antioxidant status. In this article, we provide an overview of the potential effects of Chinese Yang/Qi-invigorating tonic botanical drugs on ameliorating CKD and its complications, with particular emphasis on the possible mechanism(s) involving mitochondria-associated signaling pathways.

## Methodology

A systemic search of “PubMed”, “CNKI”, and “Google Scholar” was conducted to collect all available articles in English or Chinese that are related to the Chinese Yang/Qi-invigorating tonic botanical drugs/herbal formulations applied in the treatment of CKD, and their effects on mitochondrial function. The queries “[mitochondria (Title/Abstract)] AND [chronic kidney disease (Title/Abstract)]” were used in “Google Scholar” and “PubMed” to search for implications of mitochondrial dysfunction in CKD. To search for articles reporting the use of Chinese Yang/Qi-invigorating botanical drugs in treating CKD, the designation “Yang OR Qi OR Chinese OR botanical drug(s)”, “chronic kidney disease” (the Chinese translation for this query was used when searching in the CNKI) was used in “Google Scholar” and “CNKI”. After reading the titles and abstracts, 82 articles were selected by the reviewers to include in this article. The scientific names of all the botanical drugs were included and checked basing on the principle of scientific nomenclature for plants described in Revera et al.’s review (Rivera et al., 2014). The main mitochondrial regulatory pathways through which the Chinese Yang/Qi-invigorating botanical drugs ameliorate CKD were identified by finding the intersections and connections between the signaling pathways and molecules presented in the articles found during the aforementioned searches.

## Results

### The Implication of Mitochondrial Dysfunction in Chronic Kidney Disease

Mitochondria are vital organelles involved in the cellular regulation of energy balance and oxidative status in response to environmental stimuli. They generate bioenergy to support various cellular processes through the oxidation of fuel molecules. Furthermore, mitochondria are crucial in regulating cell survival (*via* ATP production) and death (*via* the induction of apoptosis) under conditions of stress. The kidney, which contains cells enriched with mitochondria, is an organ with high energy demand. Therefore, the maintenance of mitochondrial function plays a critical role in safeguarding renal functional integrity. The implication of mitochondrial dysfunction in association with key regulatory factors and signaling pathways involved in the pathogenesis of CKD will be discussed in the context of mitochondrial biogenesis and oxidative capacity, as well as mitochondrial dynamics.

### Mitochondrial Biogenesis and Oxidative Capacity

Mitochondria are self-replicating organelles, which contain their genome (mtDNA) and ribosomes. The regulation of mitochondrial content is controlled by cellular signals in response to various environmental stimuli. Disruption in mitochondrial biogenesis is one of the crucial processes contributing to the development of CKD.

Among the various regulatory factors, peroxisome proliferator-activated receptor γ (PPARγ) coactivator 1α (PCG-1α), the master regulator of mitochondrial biogenesis, emerges as an important component involved in the defective mitochondrial biogenesis in CKD (Sharma et al., 2013; Galvan et al., 2017). PCG-1α was found to up-regulate the expression of a large number of genes involved in mitochondrial protein synthesis and DNA replication, which results in increased mitochondrial number (Schreiber et al., 2004). Although PCG-1α is confined to the nucleus (Wu et al., 1999), it is thought to affect the replication and expression of many mitochondrial genes by interacting with a variety of proteins and stimulating downstream signaling pathways. PCG-1α co-activates two nuclear respiratory factors Nrf-1 and Nrf-2, both of which can stimulate the expression of mitochondrial transcription factor A (Tfam) (Virbasius and Scarpulla, 1994). Tfam can be translocated into mitochondria and binds to mitochondrial mtDNA, with the resultant promotion of both replication and transcription of mtDNA (Parisi and Clayton, 1991; Virbasius and Scarpulla, 1994; Wu et al., 1999). Also, PGC-1α is found to interact with estrogen-related receptor α (ERRα), which is also a major contributor to mitochondrial biogenesis (Schreiber et al., 2004).

Consistent with the crucial function of PGC-1α in mitochondrial biogenesis, the down-regulation of PGC-1α levels is prevalent in animal models of CKD and also in patients with CKD. A clinical study in diabetic patients with CKD has shown that PGC-1α levels are significantly decreased in cortical tubulointerstitial samples, which is associated with the reduction of mitochondrial proteins and mtDNA contents, an indirect indication of global suppression of mitochondrial biogenesis (Sharma et al., 2013). Furthermore, the histological features of kidney tissue were improved by the transgenic expression of PGC-1α in renal tubular cells in a mouse model of CKD (Galvan et al., 2017).

The influence of PGC-1α, a key regulator of metabolism, extends beyond mitochondrial biogenesis. Several studies have demonstrated increases in the expression of a wide array of genes related to the electron transport chain and fatty acid oxidation reactions upon the activation of PGC-1α (Wu et al., 1999; Schreiber et al., 2004). Given the marked reduction in PGC-1α levels in CKD, it seems likely that mitochondrial oxidative capacity is impaired in CKD. In support of this, Kang et al. have demonstrated decreased expression of proteins related to fatty acid oxidation, amino acid catabolism, as well as carbohydrate metabolism in kidney samples obtained from patients with CKD, with the reduction in fatty acid oxidation-related enzymes and regulators being the most prominent. The resultant increase in intracellular lipid accumulation leads to lipotoxicity, which can promote the progression of CKD (Kang et al., 2015).

One possible mechanism underlying the role of mitochondrial biogenesis in CKD was proposed by (Weinberg, 2011), wherein biogenesis allowed a fixed amount of oxidative load to be distributed among more mitochondria, thus alleviating the oxidative stress in each mitochondrion, thereby reducing the production of reactive oxygen species (ROS), with resultant protection of renal cells from oxidative injury. However, under certain conditions, such as chronic tubulointerstitial injury with pre-existing loss of the peritubular microvasculature, the unilateral enhancement of mitochondrial biogenesis could exacerbate the existing pathological conditions (Weinberg, 2011). Therefore, a more comprehensive understanding of the balance between mitochondrial biogenesis and relevant pathological processes in CKD would be required for the development of rational therapeutic interventions in CKD.

### Mitochondrial Oxidative Stress

In addition to energy generation, the mitochondrion is also the major site of cellular ROS production (Kowaltowski et al., 2009). Mitochondrial oxidative stress has long been implicated in the pathogenesis of CKD (Dounousi et al., 2006; Richter et al., 2015; Duni et al., 2019). Increased generation of ROS and impaired antioxidant status can lead to mitochondrial oxidative stress-induced kidney damage (Halliwell, 1991). Albuminuria and hyperuricemia associated with CKD may be related to enhanced mitochondrial oxidative stress (Che et al., 2014; Duni et al., 2019). For instance, albumin in cultured proximal renal tubular epithelial cells increased the extent of ROS production, which could trigger a signaling pathway leading to glomerular damage (Tang et al., 2003). Transforming growth factor (TGF)-β, a cytokine that is responsible for a wide range of renal cell pathologies, has also been shown to stimulate ROS generation in an NADPH oxidase-dependent manner in pre-glomerular vascular muscle cells (Sharma et al., 2005). Also, an increased level of ROS was detected in peripheral blood mononuclear cells of patients with stage IV-V CKD (Granata et al., 2009), implicating a general increase in oxidative stress in CKD patients.

On the other hand, mitochondrial antioxidant enzymes appeared to be commonly down-regulated in CKD, indicative of a compromised antioxidant capacity. Mitochondria are equipped with intrinsic antioxidant machinery that can scavenge excessive ROS and thus maintain a balanced oxidative status. The suppression of PGC-1α due to a deficit of endothelial nitric oxide synthase can lead to drastic decreases in activities of mitochondrial antioxidant enzymes (Weinberg, 2011). Marked decreases in activities of superoxide dismutase, catalase, and glutathione peroxidase, as well as reduced glutathione (GSH) levels, were also observed in blood samples from patients with chronic renal failure, possibly as a result of defective mitochondrial antioxidant capacity (Vaziri, 2004). The overproduction of ROS can cause a series of adverse effects including mtDNA and nuclear DNA damage, protein oxidation, mitophagy, inflammation, and cell apoptosis (Galvan et al., 2017), all of which can eventually exacerbate the progression of CKD. In this regard, treatment with antioxidants, such as omega-3 polyunsaturated fatty acids, N-acetylcysteine, vitamin E, vitamin C, and allopurinol, have all been demonstrated to produce beneficial effects in patients with renal disease (Vaziri, 2004; Che et al., 2014; Zanetti et al., 2017). Therapeutic interventions aimed at restoring a normal antioxidant status may therefore offer a promising prospect for treating or at least slowing the progression of CKD.

### Mitochondrial Dynamics

Mitochondria are dynamic organelles that constantly change their morphology, size, and quantity through fission and fusion in response to cellular signals to meet metabolic demands (Galvan et al., 2017). The balance between fission and fusion is not only crucial for the maintenance of normal mitochondrial morphology and function, but also the regulation of cellular metabolism and cell survival (Youle and Van Der Bliek, 2012). Excessive mitochondrial fission coupled with arrested fusion can result in mitochondrial fragmentation, which causes cell apoptosis and the subsequent development of CKD (Zhan et al., 2013).

Dynamin-related protein 1 (Drp1) is a protein responsible for the fission of the mitochondrial outer membrane. Multiple Drp1 molecules are recruited to the mitochondrial outer membrane by cellular signals, with the formation of a ring-like structure that subsequently severs the membrane during fission (Wai and Langer, 2016). The recruitment of Drp1 is stimulated by Rho-associated coiled coil-containing protein kinase 1 (ROCK1), which has been implicated in promoting albuminuria, mesangial matrix expansion, and podocyte apoptosis in diabetic nephropathy in mice (Wang et al., 2012a). Also, several studies have demonstrated increased mitochondrial fission and fragmentation in renal cells from mice with diabetic nephropathy (Wang et al., 2012b; Zhan et al., 2013; Zhan et al., 2015). Recent findings have shown that excessive mitochondrial fission is closely linked to the intrinsic pathway of cell apoptosis. Drp1 and several other proteins, such as Fis1 and mitochondrial E3 ubiquitin ligase MARCH5, in the mitochondrial fission machinery also appear to enhance the permeabilization of the mitochondrial outer membrane, with a resultant release of cytochrome c from the intermembrane space into the cytosol (Martinou and Youle, 2011). The released cytochrome c interacts with other proteins in the cytosol and activates caspases, resulting in cell apoptosis (Suen et al., 2008). Given the role of dysregulation of mitochondrial dynamics in inducing apoptosis of renal cells, restoration of the balance between mitochondrial fission and fusion may be beneficial in ameliorating CKD.

### Chinese Yang/Qi-Invigorating Tonic Botanical Drugs and Mitochondrial Function

#### Concepts of Yang/Qi-Invigoration in Traditional Chinese Medicine

According to ancient Chinese philosophy, everything in the Universe can be divided into two opposite but complementary aspects of Yin and Yang (Gong and Sucher, 2002; Ji and Yu, 2020). The Yin-Yang theory has been applied to the clinical practice of TCM for the diagnosis and treatment of diseases for thousands of years (Xue and Cheng, 2020). The maintenance of Yin and Yang balance is essential to promote good health in humans. Yin and Yang interaction generates Qi, which acts as the driving force of blood that carries essential nutrients and oxygen through the body (Leong and Ko, 2019). As far as body function is concerned, Qi, which is under the influence of Yang, is described as Yang/Qi in the context of health promotion (Chen et al., 2019).

Yang/Qi plays a dominant role in maintaining the normal functioning of organs in the human body, namely, the Heart, Liver, Spleen, Lung, and Kidney, according to TCM theory (Chang and Liu, 2020; Ma et al., 2020). Among these five visceral organs, the Kidney, whose functions not only include those of the kidneys in modern medicine, is of the utmost importance and is viewed as the root of Qi. Yang/Qi deficiency in the kidney can lead to chronic world-wide health problems, such as CKD, diabetes, and hypertension (Zhang, et al., 2019). The marked depletion of Yang/Qi usually causes cell death, which is consistent with the crucial role of mitochondria in determining the survival and death of the cell. As such, the mitochondrion can be viewed as the functional unit of Qi generation in cells. Yang/Qi-invigorating botanical drugs in TCM are therefore postulated to be capable of improving mitochondrial function.

### Yang/Qi-Invigorating Tonic Botanical Drugs Increase Mitochondrial Energy Generation and Antioxidant Capacity

The commonly used Yang-invigorating botanical drugs in China include *Dipsacus asper* Wall. ex C.B. Clarke (Caprifoliaceae), *Cibotium barometz* (L) J. Sm (Cibotiaceae), *Curculigo orchioides* Gaertn (Hypoxidaceae), *Epimedium sagittatum* (Sieb. et Zucc.) Maxim (Berberidaceae), *Cynomorium coccineum* subsp. *songaricum* (Rupr.) J. Léonard (Cynomoriaceae), *Cistanche deserticola* Y. C. Ma (Orobanchaceae), *Morinda officinalis* F. C. How (Rubiaceae), and *Cuscuta chinensis* Lam (Convolvulaceae). Chinese Qi-invigorating tonic botanical drugs, including *Panax ginseng* C. A. Mey (Araliaceae), *Schisandra chinensis* (Turcz.) Baill (Schisandraceae), *Astragalus mongholicus* Bunge (Leguminosae), etc., are also frequently used together with Yang-invigorating botanical drugs in the practice of TCM. Several studies have shown that Yang/Qi-invigorating tonic botanical drugs can enhance mitochondrial ATP generating capacity (ATP-GC) by increasing mitochondrial electron transport in various types of cells and tissues *in situ* and *ex vivo*, which thus delineates the biochemical basis of Yang/Qi-invigoration in TCM (Ko et al., 2006; Ko et al., 2006; Li et al., 2015). A recent study has shown that *Cistanche deserticola* Y. C. Ma (Orobanchaceae) increases mitochondrial ATP-GC in H9c2 cardiomyocytes and rat hearts by enhancing the activities of mitochondrial complex I and complex III, which indicates the possible involvement of an enhancement in oxidative phosphorylation (Ko et al., 2006; Leung and Ko, 2008). Further studies have demonstrated that Yang/Qi-invigorating tonic botanical drugs consistently enhance mitochondrial ATP-GC in H9c2 cardiomyocytes *in situ* (Leong et al., 2015), in which different mechanisms underlying the stimulation of ATP-GC are observed. The capacity of Yang-invigorating tonic botanical drugs to increase mitochondrial ATP-GC was found to be related to the fluidization of the mitochondrial inner membrane. When the mitochondrial inner membranes are stabilized by cholesterol, the stimulation of ATP-GC by Yang-invigorating tonic botanical drugs was abolished. Another representative Chinese Qi-invigorating tonic formulation, Si-Jun-Zi Decoction, consisting of *Panax ginseng* C. A. Mey (Araliaceae), *Astragalus mongholicus* Bunge (Leguminosae), *Codonopsis pilosula* (Franch) Nannf (Campanulaceae), and *Atractylodes macrocephala* Koidz (Compositae), was also shown to enhance mitochondrial energy metabolism, as evidenced by increased levels of ATP, total adenylate pool (TAP), and adenylate energy charge (ACE) in skeletal muscle of mice (Li, 2012).

Similarly, extracts of Qi-invigorating tonic botanical drugs, *Panax ginseng* C. A. Mey (Araliaceae), *Panax quinquefolius* L (Araliaceae), and *Codonopsis pilosula* (Franch) Nannf (Campanulaceae), also stimulated mitochondrial ATP-GC in cultured cardiomyocytes, with *Panax ginseng* C. A. Mey (Araliaceae) being the most potent (Wong and Ko, 2012). The stimulation of ATP-GC by Qi-invigorating tonic botanical drugs was consistently associated with an increase in cellular GSH levels (Leong et al., 2018). GSH, which serves as the first line of defense against mitochondrial oxidative stress in cells, can directly or indirectly scavenge ROS, and reverse oxidative modification of proteins, thereby preserving mitochondrial structure and function. Long-term treatment with a multi-component Yang/Qi invigorating herbal formulation (VI-28), which contains *Panax ginseng* C. A. Mey (Araliaceae), *Cordyceps sinensis* (Berk) Sacc (Ophiocordycipitaceae), *Salvia miltiorrhiza* Bunge (Lamiaceae), *Allium tuberosum* Rottl. ex Spreng (Amaryllidaceae), *Cnidium monnieri* (L). Cusson (Apiaceae), *Tetradium ruticarpum* (A.Juss) T.G.Hartley (Rutaceae) and *Kaempferia galanga* L (Zingiberaceae), was found to increase the activities of enzymatic antioxidants, such as CuZn-superoxide dismutase (SOD), and levels of non-enzymatic antioxidants, such as GSH and alpha-tocopherol (a-TOC) in rat brain, heart, liver and skeletal muscle tissues *in vivo* (Leung et al., 2005). A Qi-invigorating formulation, Bu-zhong-yi-qi decoction, was found to reduce apoptosis and necrosis in renal tubular epithelial cells and protect against 5-fluorouracil-induced renal injury in mice, possibly through an increase in mitochondrial antioxidant capacity (Xiong et al., 2016).

### Yang/Qi-Invigorating Tonic Botanical Drugs/Herbal Formulations Ameliorate Chronic Kidney Disease

In laboratories, many single Yang/Qi-invigorating botanical drugs have been shown to provide renal protective effects in animals. Red ginseng extract has been reported to protect against gentamicin-induced kidney failure by suppressing ROS production and increasing reduced glutathione levels in rats (Shin et al., 2014). Wang et al. demonstrated that treatment of a *Curculigo orchioides* Gaertn (Hypoxidaceae) extract reversed decreases in cytochrome P450 3A activity and cytochrome P450 3A4 expression in hydrocortisone-induced kidney-Yang deficiency rat models. (Wang et al., 2012b). Treatment with an *Astragalus* mongholicus Bunge (Leguminosae) and *Panax notoginseng* (Burkill) F.H.Chen (Araliaceae) formulation in combination with Bifidobacterium was found to protect kidneys in CKD by decreasing macrophage inflammatory response in the kidney and intestine *via* inhibiting Mincle signaling (Tan et al., 2020).

In the practice of TCM, a large number of Yang/Qi-invigorating tonic formulations have been shown to alleviate CKD-related symptoms, such as proteinuria, edema, and decreased glomerulus filtration. The potential pharmacological targets relevant to CKD involve a reduction in the extent of proteinuria, serum creatinine levels, urinary albumin/creatinine ratio, and urea nitrogen levels, as well as a decrease in plasma albumin levels in CKD patients. The potential pharmacological targets of Yang/Qi-invigorating tonic formulations in CKD patients are summarized in Table 1.

**TABLE 1:** Effects of Yang/Qi-invigorating formulations on the treatment of CKD.

Cause of CKD	Yang/Qi-invigorating tonic formulations	Traditional preparations of the formulations	Potential pharmacological targets relevant to CKD	References
Qi deficiency	Yu-shen recipe (*Astragalus mongholicus* Bunge (Leguminosae), *Atractylodes macrocephala* Koidz. (Compositae), *Rehmannia glutinosa* (Gaertn.) DC. (Plantaginaceae), *Alpinia oxyphylla* Miq. (Zingiberaceae), *Solanum americanum Mill.* (Solanaceae), *Dioscorea nipponica* Makino (Dioscoreaceae), *Smilax glabra* Roxb. (Smilacaceae), *Coix lacryma-jobi* var. *ma-yuen* (Rom.Caill.) Stapf (Poaceae), *Ligustrum lucidum* W.T.Aiton (Oleaceae), *Rosa laevigata* Michx. (Rosaceae), *Cornus officinalis* Siebold and Zucc. (Cornaceae), *Prunella vulgaris* L. (Lamiaceae), *Scleromitrion diffusum* (Willd) R.J.Wang (Rubiaceae), *Curcuma phaeocaulis* Valeton (Zingiberaceae), *Pseudostellaria heterophylla* (Miq) Pax (Caryophyllaceae))	Water extract	Reduce 24 h-Urinary total protein (UTP) and delay the progression of kidney disease. (A randomized clinical trial of 76 patients with chronic nephritis of Qi-yin deficiency and damp-heat syndrome aged approximately 40 years showed that 24 h-UTP, urinary erythrocyte and TCM syndrome score (including fatigue and lack of strength, limp aching lumbus and knees, etc.) were significantly decreased upon the treatment of Yu-Shen recipe. The effects showed no gender difference.)	Sun and Zhao (2020)
		To make 300 ml Yu-Shen recipe, boil the following dried herbs (proportion shown below) together in water		
		30 g *Astragalus mongholicus* Bunge (Leguminosae) root		
		15 g *Atractylodes macrocephala* Koidz. (Composita*e*)		
		15 g *Rehmannia glutinosa* (Gaertn.) DC. (Plantaginaceae)		
		15 g *Alpinia oxyphylla* Miq. (Zingiberaceae) fruit		
		15 g *Solanum americanum* Mill. (Solanaceae)		
		15 g *Dioscorea nipponica* Makino (Dioscoreaceae) root		
		15 g *Smilax glabra* Roxb. (Smilacaceae) root		
		15 g *Coix lacryma-jobi* var. *ma-yuen* (Rom.Caill.) Stapf (Poaceae)seed		
		15 g *Ligustrum lucidum* W.T.Aiton (Oleaceae) fruit		
		20 g *Rosa laevigata* Michx. (Rosaceae)		
		20 g *Cornus officinalis* Siebold & Zucc. (Cornaceae) fuit		
		20 g *Prunella vulgaris* L. (Lamiaceae) fruit cluster		
		20 g *Scleromitrion diffusum* (Willd.) R.J.Wang (Rubiaceae)		
		10 g *Curcuma phaeocaulis* Valeton (Zingiberaceae) root pre-boiled in rice vinegar		
		12 g *Pseudostellaria heterophylla* (Miq.) Pax (Caryophyllaceae)		
Yang deficiency	Shen-Qi-Wu-Ling-san (*Polyporus umbellatus* (Pers.) Fries (Polyporaceae), *Alisma plantago-aquatica* subsp. *orientale* (Sam.) Sam. (Alismataceae), *Atractylodes macrocephala* Koidz. (Compositae), *Poria cocos* (Schw.) Wolf (Polyporaceae), *Cinnamomum cassia* (L.) J. Presl (Lauraceae))	Water extract	Reduce UTP and lower serum creatinine and urea nitrogen levels. (In a randomized clinical trial, 30 patients with CKD of yang deficiency were divided into the treatment group and the control group aged approximately 36 years. The result showed that 24 h-UTP, serum creatinine and urea nitrogen levels were significantly decreased in the group with treatment of Shen-Qi-Wu-Ling-San. Insignificant association of sex and job types between two groups were demonstrated)	Wang (2019)
		To make 600 ml Shen-Qi-Wu-Ling-san, simmer the following dried herbs (proportion shown below) together in water		
		10 g *Polyporus umbellatus* (Pers.) Fries (Polyporaceae)		
		10 g *Alisma plantago-aquatica subsp. orientale* (Sam.) Sam. (Alismataceae) root		
		12 g *Atractylodes macrocephala* Koidz. (Compositae) root		
		20 g *Poria cocos* (Schw.) Wolf (Polyporaceae) sclerotium		
		10 g *Cinnamomum cassia* (L.) J. Presl (Lauraceae) twig		
Qi deficiency and dampness together with blood stasis syndrome	Shen-Yan-Yi-Hao formula (*Astragalus mongholicus* Bunge (Leguminosae), *Leonurus japonicus* Houtt. (Lamiaceae), *Dioscorea oppositifolia* L. (Dioscoreaceae), *Angelica sinensis* (Oliv.) Diels (Apiaceae), *Codonopsis pilosula* (Franch.) Nannf. (Campanulaceae))	Water extract	Reduce proteinuria and delay the progression of CKD (A randomized clinical trial of 100 patients with CKD divided into the treatment group and the control group with insignificant age and gender difference showed that proteinuria was decreased upon the treatment of Shen-Yan-Yi-Hao formula. Another clinical trial of 58 patients also showed that 24 h urine protein quantitative in the treatment group decreased more significantly than that in the control group)	Zhou et al. (2010); Zhang (2019)
		To make 150 ml Shen-Yan-Yi-Hao formula, boil the following dried herbs with 300 ml water until 150 ml liquid is left		
		30 g *Astragalus mongholicu*s Bunge (Leguminosae) root		
		30 g *Leonurus japonicus* Houtt. (Lamiaceae) (use the portion above the ground)		
		12 g *Dioscorea oppositifolia* L. (Dioscoreaceae) tuber		
		6 g *Angelica sinensis* (Oliv) Diels (Apiaceae) root		
		15 g *Codonopsis pilosula* (Franch.) Nannf. (Campanulaceae) root		
Yang deficiency	Ginger-separated Moxibustion alloy Guishenqi decoction (*Rehmannia glutinosa* (Gaertn.) DC. (Plantaginaceae), *Dioscorea oppositifolia* L. (Dioscoreaceae), Cornus officinalis Siebold & Zucc. (Cornaceae), *Alisma plantago-aquatica* subsp. Orientale (Sam.) Sam. (Alismataceae), *Poria cocos* (Schw.) Wolf (Polyporaceae), Paeonia *suffruticosa Andrews* (Paeoniaceae), *Cinnamomum cassia* (L.) J. Presl (Lauraceae) and *Aconitum carmichaelii* Debeaux (Ranunculaceae))	Water extract	Reverse abnormal changes in white blood cell and red blood cell count and reduce 24 h-UTP. (In a randomized clinical trial, after matching by sex and age, both treatment group and control group were consisting of 30 patients with CKD of yang deficiency aged approximately 46 years. The difference of sex and age between two groups was insignificant. The group with treatment of Ginger-separated Moxibustion alloy Guishenqi decoction had significant clinical efficacy in reducing 24 h-Upro, WBC and RBC)	Dai (2019)
		To make the decoction, boil the following dried herbs in 900 ml water		
		30 g *Rehmannia glutinosa* (Gaertn) DC. (Plantaginaceae) root		
		15 g *Dioscorea oppositifolia* L. (Dioscoreaceae) root		
		15 g Cornus officinalis Siebold & Zucc. (Cornaceae) fruit		
		15 g *Alisma plantago-aquatica* subsp. Orientale (Sam) Sam. (Alismataceae) root		
		15 g *Poria cocos* (Schw) Wolf (Polyporaceae) sclerotium		
		10 g *Paeonia suffruticosa* Andrews (Paeoniaceae) cortex		
		15 g *Cinnamomum cassia* (L) J. Presl (Lauraceae) twig		
		15 g *Aconitum carmichaelii* Debeaux (Ranunculaceae) root		
Qi deficiency	Si-Jun-Zi decoction (*Poria cocos (Schw) Wolf* (Polyporaceae)*, Codonopsis pilosula* (Franch) Nannf. (Campanulaceae), *Atractylodes macrocephala* Koidz. (Compositae), Glycyrrhiza uralensis Fisch. (Legumonosae)) and Six-ingredient Rehmannia decoction (*Rehmannia glutinosa* (Gaertn) DC. (Plantaginaceae), *Cornus officinalis* Siebold and Zucc. (Cornaceae), *Dioscorea oppositifolia* L. (Dioscoreaceae), *Poria cocos* (Schw.) Wolf (Polyporaceae), *Paeonia suffruticosa Andrews* (Paeoniaceae)*, Alisma plantago-aquatica subsp. orientale (Sam) Sam.* (Alismataceae))	Both are water extract To make Si-Jun-Zi decoction, boil the following dried herbs twice in 8 times water (*w/v*) for 2 h per time, and collect the supernatant.20 g *Codonopsis pilosula* (Franch) Nannf (Campanulaceae) root, 20 g *Atractylodes macrocephala* Koidz. (Compositae) root, 20 g *Poria cocos* (Schw) Wolf (Polyporaceae), 10 g *Glycyrrhiza uralensis* Fisch. (Legumonosae) root. To make Six-ingredient Rehmannia decoction boil the following dried herbs twice in 8 times water (w/v) for 2 h per time, and collect the supernatant.15 g *Rehmannia glutinosa* (Gaertn) DC. (Plantaginaceae) root, 12 g *Cornus officinalis* Siebold and Zucc. (Cornaceae) fruit, 12 g *Dioscorea oppositifolia* L. (Dioscoreaceae) root, 10 g *Poria cocos* (Schw) Wolf (Polyporaceae), 10 g *Alisma plantago-aquatica* subsp. Orientale (Sam) Sam. (Alismataceae) root, 10 g *Paeonia suffruticosa* Andrews (Paeoniaceae) cortex	Reduce levels of serum creatinine, aspartate aminotransferase, alanine aminotransferase, and lipopolysaccharide (A randomized clinical trial of 58 patients with chronic nephritis of Qi-yin deficiency aged approximately 53 years showed that the group with treatment of Si-Jun-Zi decoction had significantly reduced levels of serum creatinine, aspartate aminotransferase, alanine aminotransferase and lipopolysaccharide.)	Zhu (2018)
Qi deficiency	Yi-Ban decoction (Scrophularia ningpoensis Hemsl. (Scrophulariaceae), *Asparagus cochinchinensis* (Lour.) Merr. (Asparagaceae), *Leonurus japonicus* Houtt. (Lamiaceae), *Chrysanthemum morifolium* Ramat. (Compositae), *Astragalus mongholicus* Bunge (Leguminosae), *Isatis tinctoria* L. (Brassicaceae), *Kochia scoparia* (L) A. J. Scott (Amaranthaceae), *Scutellaria barbata* D. Don (Lamiaceae), *Glycyrrhiza uralensis* Fisch. (Legumonosae), *Patrinia scabiosifolia* link (Caprifoliaceae), *Taraxacum mongolicum* Hand.-Mazz. (Compositae), Panax quinquefolius L. (Araliaceae))	Water extractTo make Yi-Ban decoction, mix the following dried herbs according to the mass ratio of 1:1:1:1:1:1:1:1:2:2:3:3, and boil in water:*Isatis tinctoria* L. (Brassicaceae) leaf, *Kochia scoparia* (L.) A. J. Scott (Amaranthaceae) fruit, *Taraxacum mongolicum* Hand.-Mazz. (Compositae), *Patrinia scabiosifolia* link (Caprifoliaceae), *Scutellaria barbata* D. Don (Lamiaceae), *Scrophularia ningpoensis* Hemsl. (Scrophulariaceae) root, *Asparagus cochinchinensis* (Lour) Merr. (Asparagaceae) root, *Leonurus japonicus* Houtt. (Lamiaceae), *Chrysanthemum morifolium* Ramat. (Compositae) flower, *Glycyrrhiza uralensis* Fisch. (Legumonosae) root, *Astragalus mongholicus* Bunge (Leguminosae) root, *Panax quinquefolius* L. (Araliaceae) root	Reduce 24 h-UTP, urinary red blood cell count, urinary albumin creatinine ratio, serum creatinine, and urea nitrogen levels. (A randomized clinical trial of 136 patients with chronic nephritis of Qi-yin deficiency with an average of 42 years old showed that 24 h-UTP, urinary RBC, urinary albumin creatinine ratio, serum creatinine and urea nitrogen levels were reduced significantly in the group with treatment of Yi-Ban decoction. Insignificant association of sex and age between two groups were demonstrated)	Fan (2017)
Qi deficiency and heat syndrome	Er-Ban decoction (*Lobelia chinensis* Lour. (Campanulaceae), *Lonicera japonica* Thunb (Caprifoliaceae), *Forsythia suspensa* (Thunb) Vahl (Oleaceae), *Leonurus japonicus* Houtt. (Lamiaceae), *Astragalus mongholicus* Bunge (Leguminosae), *Scrophularia ningpoensis* Hemsl. (Scrophulariaceae), *Codonopsis pilosula* (Franch) Nannf. (Campanulaceae), *Scutellaria barbata* D. Don (Lamiaceae), *Ophiopogon japonicus* (Thunb) Ker Gawl. (Asparagaceae), *Kochia scoparia* (L) A. J. Scott (Amaranthaceae), *Platycodon grandiflorus* (Jacq) A.DC. (Campanulaceae), *Glycyrrhiza uralensis* Fisch. (Legumonosae))	Water extract	Reduce 24 h-UTP, urinary albumin creatinine ratio, serum creatinine, and urea nitrogen levels, and improve levels of plasma albumin. (A randomized clinical trial of 50 patients with chronic nephritis of Qi-yin deficiency and damp-heat syndrome aged approximately 39 years showed that 8 weeks of treatment of Er-Ban Decoration had significant clinical efficacy in eliminating free radicals, protecting renal cells, reducing proteinuria, dilating renal blood vessels and improving renal microcirculation. The effects showed no gender and age difference.)	Chen (2013); Wu (2015)
		To make Er-Ban decoction, mix the following dried herbs according to the mass ratio of 1:1:1:1:1:1:1:1:1:2:3:3, and boil in water		
		*Lonicera japonica* Thunb (Caprifoliaceae) flower		
		*Platycodon grandiflorus* (Jacq) A.DC. (Campanulaceae) stem and root		
		*Kochia scoparia* (L) A. J. Scott (Amaranthaceae) fruit		
		*Ophiopogon japonicus* (Thunb) Ker Gawl. (Asparagaceae) root		
		*Scutellaria barbata* D. Don (Lamiaceae)		
		*Lobelia chinensis* Lour. (Campanulaceae)		
		*Forsythia suspensa* (Thunb.) Vahl (Oleaceae) fruit		
		*Leonurus japonicus* Houtt. (Lamiaceae)		
		*Scrophularia ningpoensis* Hemsl. (Scrophulariaceae) root		
		*Glycyrrhiza uralensis* Fisch. (Legumonosae) root and stem		
		*Astragalus mongholicus* Bunge (Leguminosae) root		
		*Codonopsis pilosula* (Franch.) Nannf. (Campanulaceae) root		
Qi deficiency	Bu-Zhong-Yi-Qi decoction (*Astragalus mongholicus* Bunge (Leguminosae), *Glycyrrhiza uralensis* Fisch. (Legumonosae), *Codonopsis pilosula* (Franch.) Nannf. (Campanulaceae), *Angelica sinensis* (Oliv.) Diels (Apiaceae), *Atractylodes macrocephala* Koidz. (Compositae), *Dioscorea oppositifolia* L. (Dioscoreaceae), *Poria cocos* (Schw.) Wolf (Polyporaceae), *Actaea heracleifolia* (Kom.) J.Compton (Ranunculaceae), *Bupleurum chinense* DC. (Apiaceae), *Citrus reticulata* Blanco (Rutaceae))	Water extract	Eliminate free radicals, protect renal cells, and reduce proteinuria. Dilate renal blood vessels and improve renal microcirculation (A randomized clinical trial of 50 patients with chronic nephritis of Qi-yin deficiency and damp-heat syndrome aged approximately 38 years showed that 6 weeks of treatment of Bu-Zhong-Yi-Qi decoction had clinical efficacy in reducing 24 h-UTP, reducing RBC and improving renal function.)	Yang (2015)
		To make 200 ml Bu-Zhong-Yi-Qi decoction, boil the following dried herbs in water		
		15 g *Astragalus mongholicus* Bunge (Leguminosae) root and stem		
		6 g *Glycyrrhiza uralensis* Fisch. (Legumonosae) pre-processed using honey (to make 100 g of it, fry the root of Glycyrrhiza uralensis Fisch. (Legumonosae) in 25 g honey till no liquid remain)		
		6 g *Codonopsis pilosula* (Franch.) Nannf. (Campanulaceae) root		
		6 g *Angelica sinensis* (Oliv.) Diels (Apiaceae) root		
		6 g *Atractylodes macrocephala* Koidz. (Compositae) root		
		6 g *Dioscorea oppositifolia* L. (Dioscoreaceae) root		
		6 g *Poria cocos* (Schw.) Wolf (Polyporaceae)		
		6 g *Actaea heracleifolia* (Kom.) J.Compton (Ranunculaceae) root		
		6 g *Bupleurum chinense* DC. (Apiaceae) root		
		6 g dried pericarp of *Citrus reticulata* Blanco (Rutaceae)		
Yang deficiency	Jin-Kui-shen-Qi-Wan (*Rehmannia glutinosa* (Gaertn.) DC. (Plantaginaceae), *Cornus officinalis* Siebold and Zucc. (Cornaceae), *Dioscorea oppositifolia* L. (Dioscoreaceae), *Paeonia suffruticosa Andrews* (Paeoniaceae)*, Poria cocos (Schw) Wolf* (Polyporaceae)*, Cinnamomum cassia (L) J. Presl* (Lauraceae)*, Aconitum carmichaelii Debeaux* (Ranunculaceae))	Pill made with honey and dried herb powderEach pill weighs 9 g. To make the pill, the following dried herbs should be mixed in the ratio of 2:2:3:3:3:3:6 and grind into powder first: *Cinnamomum cassia* (L) J. Presl (Lauraceae) twig, *Paeonia suffruticosa* Andrews (Paeoniaceae) bark, *Dioscorea oppositifolia* L. (Dioscoreaceae) root, *Rehmannia glutinosa* (Gaertn.) DC. (Plantaginaceae) root, *Poria cocos* (Schw.) Wolf (Polyporaceae), *Aconitum carmichaelii* Debeaux (Ranunculaceae) root, *Cornus officinalis* Siebold and Zucc. (Cornaceae) fruit. Then, for 100 g powder, add 60–80 g honey and simmer till near solid	Normalize creatinine level, reduce proteinuria and delay the progression of kidney disease. (A randomized clinical trial of 35 patients with chronic renal failure aged approximately 36 years presented that after 3 months of treatment of Jin-Kui-Shen-Qi-Wan, creatinine level was normalized, proteinuria was reduced and the progression of kidney disease was delayed in the treatment group.)	Wang and Su (2012); Li (2018)
Yang deficiency	You-Gui Pill (*Aconitum carmichaelii* Debeaux (Ranunculaceae), *Cinnamomum cassia* (L) J. Presl (Lauraceae), *Angelica sinensis* (Oliv) Diels (Apiaceae), *Lycium barbarum* L. (Solanaceae), *Rehmannia glutinosa* (Gaertn) DC. (Plantaginaceae), *Dioscorea oppositifolia* L. (Dioscoreaceae), *Cornus officinalis* Siebold and Zucc. (Cornaceae), Cuscuta chinensis Lam. (Convolvulaceae), Eucommia ulmoides Oliv. (Eucommiaceae))	Pill made with honey and dried herb powder	Lower serum creatinine levels, reduce proteinuria, and improve glomerular filtration. (A randomized clinical trial showed that the treatment of You-Gui Pill in stage 2–3 CKD patients aged approximately 60 for 12 weeks indicated marked improvement in clinical features, including reduced serum creatinine, reduced proteinuria and improved glomerular filtration compared to the control group)	Liu et al. (2016)
		Each pill weighs 9 g. To make the pill, the following dried herbs should be mixed in the ratio of 2:2:3:3:4:4:4:4:8 and grind into powder first		
		*Aconitum carmichaelii* Debeaux (Ranunculaceae) root		
		*Cinnamomum cassia* (L.) J. Presl (Lauraceae) bark		
		*Cornus officinalis* Siebold and Zucc. (Cornaceae) fruit		
		*Angelica sinensis* (Oliv.) Diels (Apiaceae) root		
		*Dioscorea oppositifolia* L. (Dioscoreaceae) root		
		*Lycium barbarum* L. (Solanaceae) fruit		
		*Cuscuta chinensis* Lam. (Convolvulaceae) fruit		
		*Eucommia ulmoides* Oliv. (Eucommiaceae) bark		
		*Rehmannia glutinosa* (Gaertn.) DC. (Plantaginaceae) root		
		Then, for 100 g powder, add 60–80 g honey and simmer till near solid		
Qi deficiency	Jian-Pi-Yi-Qi formula (*Astragalus mongholicus* Bunge (Leguminosae), *Atractylodes macrocephala* Koidz. (Compositae), *Dioscorea oppositifolia* L. (Dioscoreaceae), *Cistanche deserticola* Y. C. Ma (Orobanchaceae), *Amomum kravanh* Pierre ex Gagnep. (Zingiberaceae), *Salvia miltiorrhiza* Bunge. (Lamiaceae), *Rheum palmatum* L. (Polygonaceae), *Glycyrrhiza glabra* L. (Leguminosae))	Water extract	Reduce serum creatinine levels, lower urea nitrogen, and urinary protein excretion. (An animal experiment with 37 nephrectomized male rats showed that administration of Jian-Pi-Yi-Qi formula for 6 weeks could improve renal function by reducing serum creatinine levels, urea nitrogen and urinary total protein levels)	Liu et al. (2018)
		Boil the following dried herbs (proportion shown below) twice in 8 times water (*w/v*) for 1 h per time, and collect the supernatant		
		30 g *Astragalus mongholicus* Bunge (Leguminosae) root		
		10 g *Atractylodes macrocephala* Koidz. (Compositae) root		
		30 g *Dioscorea oppositifolia* L. (Dioscoreaceae) root		
		10 g *Cistanche deserticola* Y. C. Ma (Orobanchaceae)		
		10 g *Amomum kravanh* Pierre ex Gagnep. (Zingiberaceae) fruit		
		15 g *Salvia miltiorrhiza* Bunge. (Lamiaceae) root		
		10 g *Rheum palmatum* L. (Polygonaceae) root		
		6 g *Glycyrrhiza glabra* L. (Leguminosae) root		

Although the therapeutic effects of Chinese Yang/Qi-invigorating tonic botanical drugs/herbal formulations are evident, the underlying biochemical mechanisms remain unclear. A clinical study has shown an association between increased oxidative stress and renal proximal tubular cell injury in CKD patients with proteinuria (Nishi et al., 2013). The decreased mitochondrial respiration was found to be associated with increased serum creatinine levels in rats (Wilson et al., 1984). The global knock-out of PGC1α, a key transcriptional regulator of mitochondrial proteins, enhanced the serum BUN and creatinine levels in mice, indicative of the important role of mitochondrial bioenergetics in the maintenance of renal integrity (Mei and Tam, 2011). Furthermore, mitochondrial dysfunction has been suggested to be involved in mediating albumin-induced renal tubular injury (Zhuang et al., 2015). Severe renal tubular structural damage and tubular cell apoptosis were observed in an albumin-challenged mouse model, which is paralleled by increases in mitochondrial ROS production and mitochondrial cytochrome c release as well as a reduction in mitochondrial DNA copy number. Given the critical role of mitochondrial dysfunction in the progression of CKD, the observation of an enhancement of mitochondrial function and antioxidant status by Chinese Yang/Qi-invigorating tonic botanical drugs/herbal formulations in both cultured cells and animal models of CKD have prompted investigation of the relevant signaling pathways.

### Effects of the Active Ingredients From Chinese Yang/Qi-Invigorating Botanical Drugs on Mitochondrial Function and Chronic Kidney Disease

To elucidate mechanisms of action, it is essential to identify the chemical components responsible for the pharmacological action(s) of Chinese Yang/Qi-invigorating tonic botanical drugs. With regard to chemical structures, components can be categorized into different groups, which include phytosterols, triterpenes, flavonoids, alkaloids, dibenzocyclooctadiene lignans, tannins, polysaccharides, and volatile oils (Pan et al., 2011). In the following section, major groups of active components identified in Chinese Yang/Qi-invigorating tonic botanical drugs and their effects on mitochondrial function and CKD will be illustrated.

#### Phytosterol and Triterpenes

**FIGURE 1:**
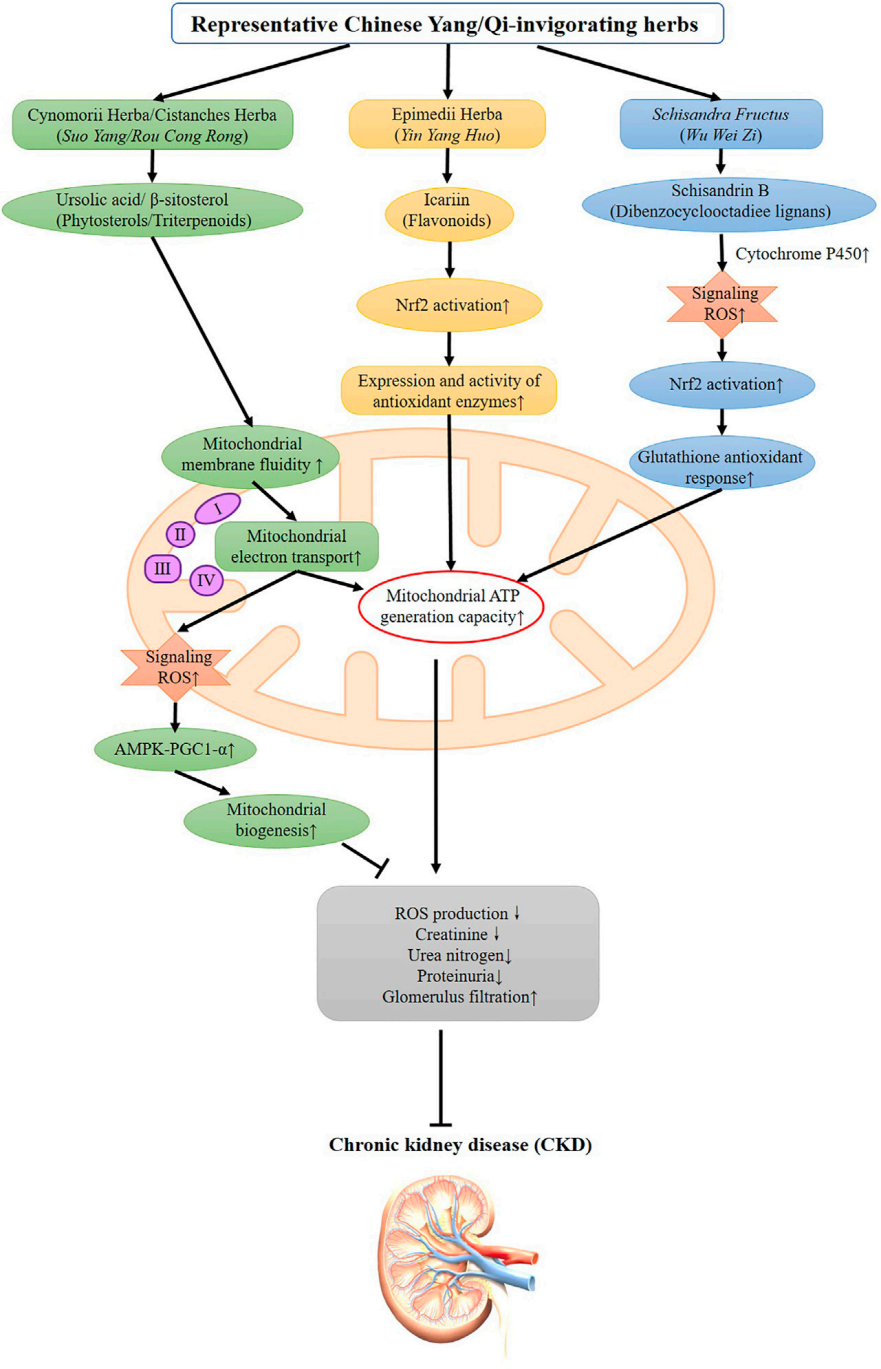
Possible signaling pathways underlying the beneficial effects of representative Chinese Yang/Qi-invigorating herbs and their active ingredients on ameliorating CKD and its complications.

Phytosterols and triterpenes are the major categories of active ingredients isolated from Chinese Yang/Qi-invigorating tonic botanical drugs (Chen et al., 2014b). An ursolic acid-rich fraction isolated from *Cynomorium coccineum* subsp. *songaricum* (Rupr.) J. Léonard (Cynomoriaceae; Cynomorii Herba), also a Chinese Yang-invigorating botanical drugs, protected against gentamicin-induced kidney damage in rats, as evidenced by the suppression in plasma creatinine and blood urea nitrogen levels (Chen et al., 2014c). However, β-sitosterol, one of the active constituents derived from *Cistanche deserticola* Y. C. Ma (Orobanchaceae; Cistanche Herba), was not shown to protect against gentamicin nephrotoxicity in rats, which was likely due to its low bioavailability in kidney tissues (Wong et al., 2014). The underlying mechanism involves the stimulation in mitochondrial ATP generation capacity as well as the enhancement of mitochondrial antioxidant status. Administration of ursolic acid was also found to ameliorate renal fibrosis in adenine-induced CKD in rats (Thakur et al., 2018). Further studies showed that both β-sitosterol and ursolic acid could incorporate into the mitochondrial inner membrane, thereby increasing membrane fluidity, with the subsequent increase in mitochondrial electron transport, as well as ATP generation (Shi et al., 2013; Chen et al., 2014a). During the process of ATP generation, a small amount of mitochondrial ROS is inevitably produced, which can serve as signaling molecules that further activate the AMPK-PGC1α pathway to increase mitochondrial biogenesis (Chen et al., 2017). (Figure 1)

#### Flavonoids

Flavonoids, another major group of active compounds from Chinese Yang/Qi-invigorating tonic botanical drugs, have also been demonstrated to protect against CKD and its related symptoms. Icariin, a flavonoid isolated from *Epimedium sagittatum* (Sieb. et Zucc.) Maxim (Berberidaceae; Epimedii Herba), was found to attenuate the progression of CKD and the loss of structural and functional integrity of kidney tissue, as evidenced by reduced serum levels of uric acid, creatinine, and blood urea nitrogen (Huang et al., 2015). Transplantation of icariin-treated human umbilical cord mesenchymal stem cells (huMSCs) was found to reduce the extent of fibrosis, oxidative damage, and inflammatory responses in renal cells in a rat model of chronic kidney failure (Li et al., 2017). A recent study has also demonstrated that oral administration of icariin can attenuate streptozotocin (STZ)-induced type 1 diabetic nephropathy, which is accompanied by a decrease in superoxide anion production and an increase in the expression and activity of antioxidant enzymes in human glomerular mesangial cells. The beneficial effect of icariin in STZ-induced type 1 diabetic nephropathy seems to be causally related to the GPER-mediated p62-dependent Keap1 degradation and Nrf2 activation (Wang et al., 2020). (Figure 1) The active ingredient of Ginseng, ginsenoside-Rd, has been found to protect against renal failure induced by cisplatin administration through a reduction in oxidative stress and an inhibition of DNA fragmentation (Yokozawa and Dong, 2001). In addition, Astragaloside IV, a lanolin alcohol-derived tetracyclic triterpene saponin isolated from *Astragalus propinquus* Schischkin (Leguminosae), was found to improve renal function and fibrosis by inhibiting miR-21-induced podocyte dedifferentiation and mesangial cell activation in diabetic mice. (Wang et al., 2018).

#### Dibenzocyclooctadiene Lignans

*Schisandra chinensis* (Turcz.) Baill (Schisandraceae), a widely used Chinese astringent botanical drug with the capability of invigorating the Qi of the five visceral organs, has been shown to produce therapeutic effects in various renal diseases (Hancke et al., 1999; Panossian and Wikman, 2008). Schisandrin B (Sch B), which is one of the major active components in *Schisandra chinensis* (Turcz.) Baill (Schisandraceae; Schisandrae Fructus), was found to protect against chemically induced nephrotoxicity through the activation of mitochondrial pathways (Chiu et al., 2008; Lai et al., 2017). A study reported by Chiu et al. showed that long-term Sch B treatment enhanced the antioxidant capacity of renal mitochondria by elevating the level and activity of antioxidant components such as GSH, α-tocopherol (α-TOC), and SOD in rats. Sch B treatment, which can elevate mitochondrial oxidative capacity and maintain mitochondrial structural integrity as well as enhance mitochondrial antioxidant capacity, protected against nephrotoxicity induced by gentamicin (Chiu et al., 2008). In addition, Sch B has also been demonstrated to ameliorate cyclosporine A (CsA)-induced nephrotoxicity in human proximal tubular epithelial cells (HK-2), presumably by suppressing the mitochondrial apoptotic pathway and preventing autophagy through the enhancement of antioxidant capacity (Lai et al., 2017). Early studies have shown that the metabolism of Sch B is mediated by cytochrome P450, with a resultant increase in ROS signaling molecules. The activation of the Nrf-2 pathway may be involved in the protection afforded by Sch B against CKD. (Figure 1).

## Discussion

Basing on our findings in this literature review, certain Chinese Yang/Qi-invigorating tonic botanical drugs/herbal formulations have shown beneficial effects on CKD *via* the mediation of mitochondrial pathways. However, there are also many other Yang/Qi-tonic botanical drugs/herbal formulations possessing the potential effects for the treatment of CKD without demonstrating the involvement of alterations in mitochondrial functions. For example, Eucommia ulmoides Oliv (EU), also known as Du-Zhong, a widely used Chinese Yang-tonic botanical drugs, was found to ameliorate renal damage in diabetic mice by decreasing advanced glycation end-products (AGEs) production and receptor of AGE expression and suppressing oxidative stress (Do et al., 2018). While the authors demonstrated that the biochemical mechanism underlying the beneficial effect of EU in diabetic nephropathy was partially mediated by the activation of the Nrf2 pathway, a more detailed mechanism of action likely involves the enhancement of mitochondrial antioxidant status. Therefore, the possible beneficial effects of more Yang- and Qi-invigorating botanical drugs/herbal formulations in the prevention and/or/treatment of CKD and the molecular mechanisms related to enhancing mitochondrial functions warrant further investigation. In addition, this paper focused mainly on the therapeutic potential of Yang/Qi-invigorating botanical drugs/herbal formulations against CKD through the modulation of mitochondrial functions in animal studies. Future clinical studies on Yang/Qi-invigorating botanical drugs/herbal formulations and effects on mitochondrial functions in CKD patients are warranted.

## Conclusion

In the practice of TCM, empirical evidence for the benefits of Chinese Yang/Qi-invigorating tonic botanical drugs/herbal formulations in the prevention/treatment of CKD has been demonstrated. Given the critical role of mitochondrial function in safeguarding renal integrity, the enhancement of mitochondrial energy metabolism and antioxidant status in kidney tissue is likely involved in renal protection. As active ingredients of distinct chemical structure exert pharmacological actions through differing signaling pathways, the effects of Chinese Yang/Qi-invigorating tonic botanical drugs/herbal formulations on CKD may result from synergistic interactions of multiple herbal components. Future studies on the biochemical and chemical basis underlying the effects of Chinese Yang/Qi-invigorating tonic botanical drugs/herbal formulations from a mitochondrial perspective will hopefully provide novel insights for the development of new drugs for the prevention and/or treatment of CKD.
